# Analyzing international clinical education practices for Canadian rehabilitation students

**DOI:** 10.1186/1472-6920-14-187

**Published:** 2014-09-09

**Authors:** Puja Ahluwalia, Debra Cameron, Lynn Cockburn, Lynn Ellwood, Brenda Mori, Stephanie A Nixon

**Affiliations:** 160-500 University Avenue, Toronto, ON M5G 1 V7 Canada

**Keywords:** Ethics, Rehabilitation, Global health, International clinical internships, Curriculum development

## Abstract

**Background:**

Clinical training in low-income countries has become increasingly popular among pre-licensure trainees from high-income countries. The Working Group on Ethics Guidelines for Global Health Training (“WEIGHT Guidelines”) were designed to identify and inform the complex and contentious field of international clinical education. The purpose of this study was to use the WEIGHT Guidelines to evaluate an international clinical internship programme for Master’s-level rehabilitation students at a Canadian university.

**Methods:**

In-depth, semi-structured interviews were conducted with eight Canadian rehabilitation researchers, educations and/or clinicians responsible for administering international internships across three clinical training programmes. Interview questions were informed by the WEIGHT Guidelines. Directed content analysis was used to identify priorities for policy, practice and research.

**Results:**

Five themes relating to strengthening international clinical education were identified: (1) from one-time internships to long-term partnerships, (2) starting a discussion about “costs”, (3) a more informed approach to student selection, (4) expanding and harmonizing pre-departure training across disciplines, and (5) investing in post-internship debriefing.

**Conclusions:**

International clinical education is fraught with ethical, pedagogical and logistical issues that require recognition and ongoing management. This is the first study to use the WEIGHT Guidelines as a qualitative research tool for assessing an existing global health education programme. Results highlight new priorities for action at the Canadian “sending institution”, including more explicit attention to the costs (broadly defined) borne by all parties. A crucial next step is deepened engagement with educational partners at the “receiving organizations” based in low-income countries to nurture dialogue regarding reciprocity, trust and sustainability of the partnership. Education research is also needed that evaluates models of pre-departure training and post-internship debriefing for trainees.

## Background

Health professional students are increasingly interested in global health education experiences [1–6]. Many universities have responded to this surge of interest by offering international clinical opportunities as part of clinical training programmes. Furthermore, the potential for international training has been viewed as a positive factor in recruitment of students to a university [7–9].

International clinical internships (ICIs) have been associated with positive personal and professional changes [7, 10–12]. ICIs have been shown to enhance flexibility, cultural sensitivity, confidence, clinical skills, and cross-cultural communication [7, 10–14]. ICIs have been linked with future practice patterns, including work in public health, multi-cultural settings and underserviced areas within local community [7, 10, 14].

Despite these potential benefits, when ICIs occur in low or middle income countries, they have been critiqued for increasing the burden on low and middle income country (LMIC) partners, and for creating harms when students or faculty have insufficient awareness of cultural norms in the host community [1, 4, 15]. Physiotherapy and occupational therapy students have found that differing cultural belief systems encountered on ICIs can be difficult to navigate without appropriate pre-departure preparation and onsite support [11, 12, 16]. ICIs can be time-consuming for faculty in terms of developing partnerships and ensuring appropriate supervision for students [16, 17].

Despite the rapid rise of global health training programmes across North America, there is little research evaluating programme processes. There is no uniform approach for organizing ICIs, there is little infrastructure to assist student planning, and the process is often viewed as ad hoc [18, 19]. As such, there have been multiple appeals for increased evaluation of how ICIs are organized and implemented to ensure better outcomes for students and more equitable engagement with partners in LMICs [4, 7, 20, 21].

The “WEIGHT Guidelines”, developed by the Working Group on Ethics Guidelines for Global Health Training, are designed to address a variety of global health issues involving the sending institutions (e.g., North American University), host institutions (e.g., the clinical training site in an LMIC), trainees (e.g. North American students), mentors (e.g., clinical supervisors) and donors (e.g., those resourcing the educational opportunities) [22]. Through a literature review and consultation among its 13 expert members, the WEIGHT Guidelines were developed to provide guidance on mitigating ethical issues and promoting best practices for implementing global health initiatives [22]. The Guidelines offer a comprehensive framework for ethical reflection, but have not yet been used in research evaluating global health or rehabilitation education.

The purpose of this study was to use the WEIGHT Guidelines to investigate an international clinical internship programme for occupational therapy (OT), physiotherapy (PT), and speech-language pathology (SLP) students at a Canadian university. In particular, we explored the current models and processes being used by the sending organizations, the benefits and limitations of these approaches, and perceptions of the priorities for practice and research.

## Methods

### Study design and setting

This qualitative, descriptive study was conducted at the Rehabilitation Sciences Sector (RSS) at the University of Toronto, which houses Master’s-level clinical training programmes in OT, PT and SLP. The curriculum of each program includes multiple 5–10 week internships at clinical training sites, which may include sites in LMICs. Most of the ICIs are conducted at clinical training sites facilitated by the International Centre for Disability and Rehabilitation (ICDR), which include: a rehabilitation programme in Cameroon; a rural rehabilitation clinic in Kenya, a peri-urban hospital in the Philippines, an academic hospital and a community-based rehabilitation programme in Tanzania, and a school for children and vocational centre for adults with disabilities in Trinidad. ICDR was established in 2004 at the University of Toronto to advance research and education in the rehabilitation sciences that improves the lives of people with disabilities globally [23]. Each of ICDR’s international partnerships is supported by a formalized ICDR-Group that nurtures and sustains the partnership over time. Since 2000, over 150 students from the RSS have conducted ICIs at ICDR sites.

This study adheres to RATS guidelines for reporting qualitative studies [24].

### Participants and recruitment

Participants were eligible for the study if they were affiliated with the RSS and/or ICDR, and had insight and experience facilitating ICIs within the RSS. The scope of the current study did not include education partners at host institutions or students who had experienced ICIs, although this is a priority for a later phase of research. Potential participants were invited to participate by email. Ethics approval was obtained by the University of Toronto Research Ethics Board.

### Data collection

In-depth semi-structured interviews were conducted in May and June 2012. Interviews ranged from 30 minutes to 3.5 hours. The 3.5 hour interview was performed over two sessions whereas all the others were conducted in one session. Interviews were performed in person, by phone or by Skype. Interviews were digitally recorded and fieldnotes were written throughout and following each interview. Relevant portions of interviews were transcribed verbatim. The interview guide was based on the WEIGHT Guidelines [22]. Guideline categories were combined and/or re-ordered to better enable a coherent dialogue. The interview guide was revised after the first two interviews to further improve flow and reduce repetition of concepts (see Table 1).

**Table 1:** **Interview guide based on the WEIGHT guidelines**

Questions for each guideline topic:	
For each of the following guidelines regarding international clinical internationals:	- What is your *current approach* (how do you do this)?
	- In what ways does this *work well*?
	- In what ways does this *not work well*?
	- How do you think it *ought to work* in an ideal scenario?
	- Who exactly is *responsible* for what aspects of this?
	- Is there anything we have not discussed that you would like to add?
**Guidelines topics:**	
1. **Toward equitable benefits:** Develop well-structured programs so that host and sender derive mutual, equitable benefits, including:	a. **Written agreement:** discuss expectations and responsibilities of both host and sending institutions and agree on terms before program implementation (may be outlined in MOU), and revisit expectations on a periodic basis
	b. **Local priorities:** consider local needs and priorities regarding the structure of the ICI
	c. **Recognizing all costs:** recognize the true cost to all institutions (e.g., costs of orientation, insurance, translation, supervision and mentoring, transportation, lodging, health care, administration) and ensure they are appropriately reimbursed and/or **Monitoring costs:** Monitor costs and benefits to host institutions, local trainees, patients, communities, and sponsoring institutions to assure equity
	d. **Nesting in long-term relationships:** aspire to maintain long-term relationships so that short-term experiences may be nested within them
	e. **Transparent motives:** promote transparency regarding the motivations for establishing and maintaining programmes (e.g., to meet an educational mission, to establish a relationship that might be used to support research, to meet student need) and identifying and addressing any conflicts of interest or conflicts of engagement (e.g., to local patients, communities, or local trainees compared with global health trainees) that may arise from such a programme
2. **Explicit agreements:** Clarify goals, expectations and responsibilities through explicit agreements and periodic review by:	a. Senders and hosts
	b. Trainees and mentors
	c. Sponsors and recipients
3. **Training for all involved:** Develop, implement, regularly update and improve formal training for trainees and mentors, both local and foreign, regarding materials that includes:	a. Norms of professionalism (local and sending)
	b. Standards of practice (local and sending)
	c. Cultural competencies e.g., behaviours (local and sending)
	d. Dealing appropriately with conflicts (i.e. professionalism, culture, scientific and clinical differences of approach)
	e. Language capability
	f. Personal safety and Safety of Trainees: Promote safety of trainees to the extent possible (e.g., vaccinations, personal behaviours, medications, physical barriers, security awareness, road safety, sexual harassment, psychological support, insurance and knowledge of relevant local laws)
	g. Implications of differential access to resources for foreign and local trainees
4. **Conflict resolution:** Encourage non-threatening communication to resolve ethical conflicts as they arise in real-time and identify a mechanism to involve the host and sending institutions when issues are not readily resolved	
5. **Clarifying trainee’s abilities:** Clarify the trainees’ level of training and experience for the host institution so that appropriate activities are assigned and patient care and community well-being is not compromised	
6. **Selection of trainees:** Select trainees who are adaptable, motivated to address global health issues, sensitive to local priorities, willing to listen and learn, whose abilities and experience matches the expectations of the position, and who will be good representatives of their home institution and country	
7. **Supervisory model:** Establish effective supervision and mentorship of trainees by the host and sending institution, including the selection of appropriate mentors and supervisors and facilitating communication among them.	
8. **Feedback from trainees:** Establish methods to solicit feedback from the trainees both during and on completion of the program, including exit interviews, and track the participants post-training to evaluate the impact of the experience.	

### Data analysis

Interview data were coded according to the WEIGHT Guidelines as outined in the interview guide shown in Table 1. We then conducted descriptive analysis *within* each WEIGHT category to present participants’ perceptions, and the degree of consensus or divergence around various issues [25]. We then analyzed these descriptive findings *across* categories to surface the five higher-level patterns that are presented below. Additional collaborative research team meetings were conducted to reflect on implications of the findings for future practice and research within the RSS and ICDR.

## Results

### Participants

Nine individuals holding 10 positions within the RSS and/or ICDR were invited to participate in this study. All but one agreed, so the final sample included eight participants. Five participants held paid or volunteer positions with ICDR. Five of the participants were faculty within the RSS at the University of Toronto. All participants were rehabilitation professionals (two OT, three PT, three SLP).

### Priorities for strengthening international clinical internships

**Figure 1:**
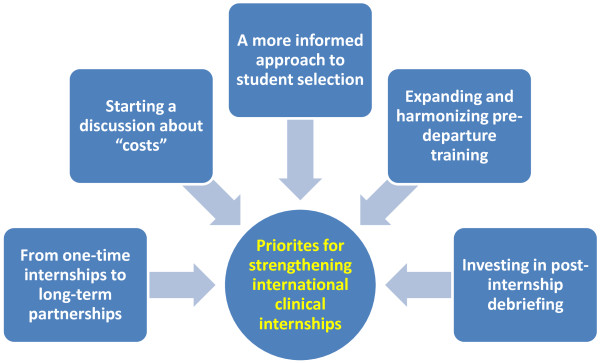
**The 5 themes for strengthening international clinical internships.** This figure represents the five main themes that should be considered as priorities for strengthening international clinical internships. The themes are as follows: From one-time internships to long-term partnerships; Starting a discussion about ‘costs’; A more informed approach to student selection; Expanding and harmonizing pre-departure training; Investing in post-internship debriefing.

Five themes emerged as priority areas for attention within the ICI programme (see Figure 1).

#### From one-time internships to long-term partnerships

Participants agreed that, in principle, ICIs should be one component within a broader, long-term relationship between the host and sending institutions. Participants viewed this approach as essential for more equitable sharing of burdens and benefits, improved student experiences, and enhanced sustainability of the clinical training partnership. Participants also noted that this approach makes ICIs easier to organize.

However, participants acknowledged that achieving this level of maturity in an international partnership is challenging and requires significant investment of time and personal relationship-building. In all cases, the long-term international partnerships described by participants were the result of one or more Canadians nurturing relationships with individuals at the host institution over many years. This model is in contrast to partnerships formed first at the institutional level within which personal ties are then created. Participants largely perceived the result of the current approach to be more “authentic partnerships” based on trust and mutual understanding. However, these interpersonal relationships were largely informal and, thus, lacking formalized agreements (beyond the departmental placement agreements) or wider institutional support. Several participants noted the vulnerability of such partnerships when they are based on individual relationships. As one participant explained:

*Really, the only explicit agreement that we have in place, and this is probably a bit of a problem… is a [internship] contract between UofT and the host institution. That is totally related to student fieldwork placements and it’s totally related at this point to what the arrangement is for our students going there… For sort of all the other stuff that you have agreements about, most of that is very informal. Most of it is, you know, email exchanges or verbal exchanges or history that’s built up, precedent, what do the students usually do, how do they usually do it, etc., etc., a lot of that is not written down.*

Most participants recommended formalizing partnerships through creating a memorandum of understanding or other mechanism for articulating aims, expectations and responsibilities of all partners. At present, the only required documentation is a “placement agreement”, which describes the host’s responsibilities for ensuring the adequate education and safety of students. One participant warned that while a wider agreement would be useful, LMIC partners might not have time to engage in such a process given their other demands.

It is also noteworthy that not all participants agreed that long-term partnerships should be the only option for international education, and that one-off internships should be an option for students to allow for greater flexibility in training. Concern was also raised regarding how to begin and end relationships related to international clinical training.

#### Starting a discussion about “costs”

All participants acknowledged the wide range of monetary and non-monetary costs incurred by the sending institutions, host institutions, and students during the planning and implementation of ICIs. Participants also noted that such costs were rarely discussed and, in many cases, can be challenging to track. They explained that not only are these costs difficult to calculate, but they were also perceived as “impossible to reimburse” within current funding models. When asked about costs borne by her partners at the host institution, one participant stated:

*From the host institution [point of view], do we track that at all? Really no, other than this increasing recognition that it costs them a lot.*

Participants reported attempts to mitigate host costs in various ways, including student placement fees, equipment donations, and fundraising by Canadian partners. Cost to students varied across the rehabilitation departments, with some departments able to offer scholarships. However, all students were required to take responsibility for most costs related to their international training experience. As a result, ability-to-pay became a prerequisite for student participation in these learning experiences.

Departments experienced different costs depending on the supervisory model for different professions and at the different sites. For example, one model required that a Canadian supervisor was funded to travel with the students because there was no local supervisory support available. In other models, however, supervision was provided to Canadian students by local host professionals, which shifted costs incurred from sender to host. It is noteworthy that participants viewed the multiple models in place as relatively unproblematic given that each of the international training partnerships had been developed independently and iteratively over time. Participants saw the value in moving toward a more harmonized approach to internships at the different sites, but viewed resource constraints as the major obstacle. Furthermore, in many cases, the flexibility of the various supervisory models was viewed as a strength for the university’s training programme.

#### A more informed approach to student selection

Guidelines for selecting students for ICIs were viewed by participants as an area that had received insufficient attention. Participants reported that student selection processes varied according to profession and clinical education site. However, all agreed that the current approach begins with students self-selecting based upon their motivation to apply for an ICI. Beyond this initial step, there was inconsistency among some participants regarding subsequent selection procedures, which resulted in concerns as noted by this participant:

*“I guess the thing is, is, there’s really no screening mechanism that we have in place to really understand if the student fully comprehends or appreciates the context in which they’re going and will represent, I mean, will represent the [sending organization] well.”*

Participants viewed a more comprehensive approach to student selection as ideal, but cited constraints related to time and coordination as prohibiting development of an explicit and harmonized approach. Most participants also viewed the current process as working “well enough” despite its shortcomings.

#### Expanding and harmonizing pre-departure training

All participants were involved with pre-departure training for students in some way; however, there was consensus that a more robust and coherent approach would be in service of both students and those providing this service. All students are required to participate in a university-wide safety session, which provides general training for overseas student travel. However, participants argued the need for additional preparation focused on details of the training site (e.g., logistics of accommodation and local travel). Several participants explained the importance of discipline-specific training to ensure students understand training expectations as set out by their respective professional licensing body.

Various participants raised the need for pre-departure training that helped students understand critical theoretical perspectives on global health and international development, including post-colonial theory, cultural competence, and reflexivity. These topics were described as crucially important for students participating in clinical training in new settings, but also challenging to convey in the context of clinical training programmes. One participant raised the need for training related to social media and the potentially damaging impact that student media posts can have on host and sending institutions given the blurred line between the private and public spheres. Furthermore, several participants emphasized the need for students to ensure appropriate behaviour after clinic hours as guests in the community.

Participants advocated for investment into a multi-faceted pre-departure training curriculum that was centrally coordinated and took advantage of the teaching abilities of various stakeholders, including students who have completed internships.

#### Investing in post-internship debriefing

All participants discussed the importance of post-internship debriefing because of its role in helping students deal with reverse culture shock. Debriefing was also viewed as an important part of relationship-building, whereby the student could share strengths and limitations of the learning experience, which could then be addressed by the educational partners. Debriefing was also viewed as a mechanism for engaging interested students to become part of the international partnership team.

Most participants expressed concern that the current system is only partially effective. When asked about engaging with students following internships, one participant noted:

*That’s the piece that we don’t do a very good job of. Summarizing what we’ve learned. We would do some of that, but it’s in bits and pieces.*

Several participants explained that debriefing is difficult to operationalize since it is not a formal requirement for students. In many cases, students undertook ICIs as their final clinical requirement, which meant that they were no longer in the pre-licensure programme following the international experience and may not return to the university. Participants largely agreed that a formal post-internship debriefing programme should be developed, and that participation should become a mandated expectation of students undertaking ICIs.

## Discussion

Given the increased recognition of ethical, pedagogical and logistical challenges associated with international clinical education, there is a growing literature providing guidance in the form of recommendations [3, 4, 22] or by offering anecdotal reflection on personal experiences [15, 20]. This is the first article to present an empirical study that uses the WEIGHT Guidelines to examine an international clinical education programme for Master’s-level OT, PT and SLP students based at a university in Canada. Results affirm the importance of key programme components (e.g., pre-departure training and post-internship debriefing) and offer deeper analysis of the complexity of operationalizing global health education in ethical and sustainable ways (see Table 2).

**Table 2:** **Key messages from this study**

**Implications for global health internship programmes:**	The main barrier to implementing preferred or ideal models of ethical and sustainable global health internships was investment in adequate human resources to develop and coordinate more coherent systems.
	Comprehensive programmes of pre-departure training and post-internship debriefing are crucial, and require teaching faculty to have skill sets in diverse content areas (e.g., debates in international development, specific knowledge of training site, profession-specific knowledge) and multiple forms of knowledge (e.g., factual, reflexive).
	Partnership development with educators at host institutions requires not only the sustained commitment of time by Canadian faculty (typically over multiple years) but also particular skills and insights related to global health ethics.
**Implications for future research:**	The WEIGHT Guidelines may be adapted for use as an interview guide for qualitative studies exploring best practices related to the ethics of global health activities (see Table 1).
	Metrics are required to make explicit the costs (including financial, human resource, time diverted from clinical care, etc.) incurred by all parties in global health education collaborations. This is not yet common practice, but is a crucial step toward transparency and equity.
	Researchers must critically reflect on the limitations of common research methods for understanding perspectives and realities in settings very different than their own (e.g., for host partners). Alterative paradigms (e.g., postcolonial approaches) may have much to offer.

Across the five themes, a recurring finding was the perception that the main barrier (and in some cases, the single barrier) to realization of a preferred or even ideal model was investment in adequate human resources to develop and coordinate more coherent systems. The extent of required investment will vary and may be relatively small depending on the institutional priority given to global health education.

The five priorities for programme development were clear, and have a basis in the literature as well as this focused inquiry. Pre-departure training and post-internship debriefing are well-understood as necessary for enhancing cultural competence and sensitivity, and for evaluating the education experience [20, 26–28]. However, these are complex pedagogical processes engaging diverse content areas (e.g., debates in international development, specific knowledge of training site, profession-specific knowledge) [29] and multiple forms of knowledge (e.g., factual, reflexive) [30, 31]. As such, faculty will require multiple skillsets to develop and evaluate pre-departure training and post-internship educational programmes.

Similarly, partnership-development skills are required among the faculty responsible for maturing international training experiences into sustainable and equitable global health partnerships. Development of informal and formalized agreements is an important but challenging component of this process, which can be particularly complex in larger organizations [16, 32, 33]. The explicit recognition of costs incurred by all parties surfaced as another priority for the programme in this study, and may be understood as synergistic with the broader move toward improved communication and partnership development [2,14,17,28].

### The WEIGHT guidelines as a research framework

This was the first study to use the WEIGHT Guidelines [22] as a conceptual lens for a qualitative analysis of global health education practices. In particular, we used the Guidelines as the basis for the interview guide in data collection, and the Guideline categories as the deductively-derived “codes” for our coding framework during data analysis.

Overall, the WEIGHT Guidelines proved to offer a useful framework for questioning current processes and ethical concerns that may occur in organizing an ICI. In many cases, questions raised by the Guidelines represented areas or issues not yet considered by participants in their global health education work. As such, the Guidelines prompted reflection on a more comprehensive range of issues than would have been the case if the study had not used this lens. We note that the issues prioritized in the Guidelines are not distinct to rehabilitation, and will be equally relevant for other internship programmes related to global health (see Table 2). However, we also found the Guidelines to be repetitive (e.g., multiple concepts related to costs). As such, a contribution of this study is the synthesized interview guide (see Table 1) that provides the basis for a coherent interview while ensuring all Guideline areas are included.

### Limitations

This study exclusively sought the perspectives of individuals involved in ICI coordination at the Canadian sending institution. As such, results must not be interpreted as reflecting the perspectives of students or of education partners at the host institutions at our fieldwork sites, whose views are crucially important but were not explored in this inquiry.

A second limitation is the pre-existing collegial relationships that may have existed between members of the research team and the participants. These sorts of pre-existing relationships can lead to potential participants feeling coerced to participate or hesitant to share critical perspectives during interviews for fear of jeopardizing working relationships. We sought to mitigate these challenges by explicitly acknowledging these potential concerns verbally and in the consent form, and emphasizing for participants that the aim of the inquiry was to better understand the shortcomings as well as successes of the programme.

### Implications for future research

Three priorities for research emerged in this study. First, research to identify and develop strategies and metrics for quantifying the wide range of costs and benefits associated with global health education for *all* partners is crucial for ensuring more equitable sharing of costs and benefits among partners.

Second, obtaining a better understanding of the perspectives of other stakeholders, especially host partners and clients, is an essential area for future research. However, we agree with Hanson who cautions about the limitations of commonly-used research approaches (e.g., surveys, interviews) for developing a sophisticated and authentic understanding of the perspectives of LMIC stakeholders regarding their role in educating high income country students [30, 31]. Our ability to conduct research with education partners at our own sending institutions (i.e., given our understanding of the cultural and professional systems in Canada, given our training and lives within these same systems) must not lead to the conclusion that we are well-positioned to use the same methods to conduct research with education partners in LMICs (i.e., whose historic and contemporary cultural and professional experiences may be so different than our own). Consideration must be given to theoretical (e.g., contributions of postcolonialism) and methodological (e.g., community-engaged participatory research) issues underpinning inquiries led by partners at sending institutions into experiences of “receiving partners” within global health education programmes. Furthermore, research conducted by educational partners in LMICs that does not involve high income country partners should be considered. As a first step in this direction, several of the authors have undertaken a participatory inquiry with education partners in Cameroon regarding the contributions of post-colonial theory to thinking about rehabilitation with children with disabilities.

Finally, given the importance and the complexity of pre-departure training and post-internship debriefing, educational research should be conducted that pilots and scales up this programming.

## Conclusions

International clinical education is fraught with ethical, pedagogical and logistical issues that require recognition and ongoing management. This is the first study to use the WEIGHT Guidelines as a qualitative research tool for assessing an existing global health education programme. Results highlight new priorities for action at the Canadian “sending institution”, including more explicit attention to the costs (broadly defined) borne by all parties. A crucial next step is deepened engagement with educational partners at the “receiving organizations” based in low-income countries to nurture dialogue regarding reciprocity, trust and sustainability of the partnership. Education research is also needed that evaluates models of pre-departure training and post-internship debriefing for trainees.

## Abbreviations

ICDR: International centre for disability and rehabilitation
ICI: International clinical internship
LMIC: Low- or middle-income country
OT: Occupational therapy
PT: Physiotherapy
RSS: Rehabilitation sciences sector
SLP: Speech-language pathology
UofT: University of Toronto
WEIGHT: Working group on ethics guidelines for global health training.
